# α-Ketoglutarate alleviates osteoarthritis by inhibiting ferroptosis via the ETV4/SLC7A11/GPX4 signaling pathway

**DOI:** 10.1186/s11658-024-00605-6

**Published:** 2024-06-14

**Authors:** Rong He, Yuchi Wei, Zeyu Peng, Jie Yang, Zhenwei Zhou, Ailin Li, Yongji Wu, Mingyue Wang, Xiangyan Li, Daqing Zhao, Zhonghua Liu, Haisi Dong, Xiangyang Leng

**Affiliations:** 1 College of Traditional Chinese Medicine, Changchun University of Chinese Medicine, Changchun, 130117 Jilin Province China; 2 Northeast Asia Research Institute of Traditional Chinese Medicine, Changchun University of Chinese Medicine, Changchun, 130117 Jilin Province China; 3 Department of orthopaedics, The Third Affiliated Hospital of Changchun University of Chinese Medicine, Changchun, 130117 Jilin Province China

**Keywords:** Osteoarthritis, α-ketoglutarate, Ferroptosis, ETV4/SLC7A11/GPX4 signaling pathway

## Abstract

Osteoarthritis (OA) is the most common degenerative joint disorder that causes disability in aged individuals, caused by functional and structural alterations of the knee joint. To investigate whether metabolic drivers might be harnessed to promote cartilage repair, a liquid chromatography–mass spectrometry (LC–MS) untargeted metabolomics approach was carried out to screen serum biomarkers in osteoarthritic rats. Based on the correlation analyses, α-ketoglutarate (α-KG) has been demonstrated to have antioxidant and anti-inflammatory properties in various diseases. These properties make α-KG a prime candidate for further investigation of OA. Experimental results indicate that α-KG significantly inhibited H_2_O_2_-induced cartilage cell matrix degradation and apoptosis, reduced levels of reactive oxygen species (ROS) and malondialdehyde (MDA), increased superoxide dismutase (SOD) and glutathione (GSH)/glutathione disulfide (GSSG) levels, and upregulated the expression of ETV4, SLC7A11 and GPX4. Further mechanistic studies observed that α-KG, like Ferrostatin-1 (Fer-1), effectively alleviated Erastin-induced apoptosis and ECM degradation. α-KG and Fer-1 upregulated ETV4, SLC7A11, and GPX4 at the mRNA and protein levels, decreased ferrous ion (Fe^2+^) accumulation, and preserved mitochondrial membrane potential (MMP) in ATDC5 cells. In vivo, α-KG treatment inhibited ferroptosis in OA rats by activating the ETV4/SLC7A11/GPX4 pathway. Thus, these findings indicate that α-KG inhibits ferroptosis via the ETV4/SLC7A11/GPX4 signaling pathway, thereby alleviating OA. These observations suggest that α-KG exhibits potential therapeutic properties for the treatment and prevention of OA, thereby having potential clinical applications in the future.

**Supplementary Information:**

The online version contains supplementary material available at 10.1186/s11658-024-00605-6.

## Introduction

All joint tissues may be affected by osteoarthritis (OA), which causes degenerative changes in joints and the surrounding tissues, resulting in persistent pain, stiffness, joint deformity, joint dysfunction [1]. The prevalence of OA has greatly increased worldwide due to population aging. A 2017 study found that the age-standardized incidence of OA varied from 2090.3 to 6128.1 cases per 100,000 people [2]. Projections indicate an increase in OA incidence from 26.6% to 29.5% by 2032 [3]. The economic cost of OA to society and healthcare systems is substantial [4].

Articular cartilage is particularly vulnerable to the effects of OA and undergoes significant degradation during this process [5]. Chondrocyte death and an imbalance in extracellular matrix (ECM) metabolism are key factors in OA pathogenesis [6]. Research has demonstrated that the induction of oxidative stress contributes to chondrocyte ferroptosis [7], ECM degradation, and the exacerbation of OA through the suppression of anabolic gene expression, notably including SOX9, COL2A1, and ACAN [8, 9].

Ferroptosis represents a distinct mode of cellular demise, characterized by iron-catalyzed aberrations in lipid peroxide metabolism. This process escalates the generation of reactive oxygen species (ROS), precipitates lipid peroxidation, and culminates in cellular mortality [10, 11]. Recent investigations have identified the presence of iron accumulation and proteins associated with ferroptosis in the serum, plasma, synovial fluid, and articular cartilage of individuals afflicted by OA [12, 13]. This correlation has been corroborated by both animal and cellular research [14, 15]. Chondrocyte ferroptosis has been identified as a key contributor to OA. In OA rats, decreased levels of the crucial ferroptosis regulators, glutathione peroxidase 4 (GPX4) and solute carrier family 7 member 11 (SLC7A11), have led to enhanced cartilage ferroptosis [14, 16]. Notably, inhibiting chondrocyte ferroptosis in OA rats has been shown to significantly improve joint function [17, 18], suggesting that inhibiting ferroptosis is a promising treatment strategy for OA.

α-KG, which is essential in almost all organisms, plays pivotal roles in antioxidation [19–21], anti-inflammation [22, 23], and collagen synthesis [24, 25]. Studies indicate that α-KG enhances lipid metabolism and protects against lipid peroxidation damage [15]. In a study on the occurrence of liver cancer, taking α-KG increased the level of glutathione (GSH) and its dependent enzyme activity, and reversed the increase of lipid peroxide level caused by carcinogenic treatment of N-nitrosodiethylamine and carbon tetrachloride [26, 27]. Recent research has indicated α-KG attenuates OA through the enhancement of mitochondrial autophagy and the suppression of ROS synthesis [28]. Our research has demonstrated that α-KG mitigates oxidative stress and ferroptosis, thereby alleviating OA.

In the current research endeavor, we investigated the therapeutic effects of α-KG on OA both in vivo and in vitro through the inhibition of ferroptosis. Our data showed that α-KG exhibits potent anti-osteoarthritic activity, much of which can be attributed to its capacity to ward off ferroptosis. These findings reveal a new and unique mechanism of α-KG treatment in OA, which has significant clinical and therapeutic implications.

## Materials and methods

### Animal models and drug administration

The Institutional Animal Care and Use Committee at Changchun University of Chinese Medicine approved this work (Approval No. 2022145, 12/3/2022). To establish the OA model, rats were anesthetized with 3% sodium pentobarbital; thereafter, the right knee was surgically opened to perform the destabilization of the medial meniscus (DMM), avoiding damage to surrounding tissues [29, 30]. The DMM rats were allowed to run on a horizontal treadmill five times a week at an average speed of 20 m per minute. The points at 1 week and 8 weeks represent the early and late stages of OA, respectively [31–34]. Eight-week-old male Sprague‒Dawley rats were randomly assigned to the Blank group (*n* = 8) or Model group (*n* = 8) to study serum metabolic alterations. To assess the therapeutic effects of α-KG in vivo, rats were randomized into four groups: the Blank group (*n* = 10), the Model group (*n* = 10), the α-KG group (*n* = 10), and the Positive Control (PC) group (*n* = 10). Celebrex served as the PC drug. In the α-KG group, 100 μL of α-KG (0.32 mM) was injected weekly into the right joint after 1 week of DMM. The Model group was injected with phosphate-buffered saline (PBS). The PC group received daily gavage of celebrex (12 mg/kg). Samples were collected 8 weeks after DMM. Based on relevant experimental evidence, we chose the α-KG derivative DM-α-KG (Sigma, USA) for our in vitro and in vivo studies [28, 35].

### Collection of serum and knee cartilage

Rats were anesthetized with 3% sodium pentobarbital following a 14-h fast. Subsequently, articular cartilage was harvested from the knee joints. Blood samples were collected from the abdominal aorta of the rats using a blood collection needle, and 5 mL of blood was collected from each rat. Serum was isolated through centrifugation of blood at 3,000 rpm for 15 min and subsequently preserved at -80°C for future applications.

### Serum metabolomics

A 100 μL serum sample was combined with 400 μL of 80% methanol solution in an EP tube, incubated in an ice bath for 5 min, and then centrifuged at 4 °C and 15,000×*g* for 20 min. The supernatant was diluted with mass spectrometry-grade water to achieve a 53% methanol concentration and then centrifuged at 4 °C and 15,000×*g* for 20 min. The resulting supernatant was collected for liquid chromatography–mass spectrometry (LC–MS) analysis [36]. Multivariate statistical techniques, specifically principal component analysis (PCA) and partial least squares-discriminant analysis (PLS-DA), were used to examine the metabolites. With VIP > 1.0, FC > 1.2, or FC < 0.833 and P value < 0.05, the differentially abundant metabolites were screened out [37–39], and volcano maps and heatmaps were drawn on Bioinformatics (http://www.bioinformatics.com.cn/).

### Detection of the α-KG level

Take out the frozen knee joint tissue and delicately scrape off the cartilage from the tibia and femur. The cartilage was placed in a mortar, ground, and liquid nitrogen was periodically added to maintain a low temperature until it was powdered. The quantification of α-KG levels in both the serum and articular cartilage samples from the rats was achieved utilizing an Enzyme-linked immunosorbent assay (ELISA) kit (Mlbio, China).

### Weight-bearing test and gait analysis

Pain levels were assessed by quantifying hind paw weight-bearing time using the dynamic weight bearing test (BIO-DWB2-M, USA). Initially, the rats were weighed, and their weights were recorded. Subsequently, the environmental adaptation lasted 10 min, while the sensor reception period was 2 min. The program commenced once the rats were placed on the load-bearing sensors. Weight-bearing times were assessed by the cumulative duration of contact of each paw with the ground. Tests were conducted weekly posttreatment.

The DigiGait™ (Mouse Specifics Inc., USA) allows rats to walk on a motorized transparent treadmill belt, with the animals' gait being recorded by a camera underneath the transparent belt. After manual cropping and importing, the DigiGait™ system automatically analyzes the video [40]. The system automatically calculates the landing area of the right hind paw and the differences in weight-bearing between the left and right hind paws throughout the gait cycle. Gait analyses were conducted every two weeks after treatment.

### Micro-CT analysis

Following muscle tissue removal, each rat's leg bone was scanned and analyzed using Micro-CT (PerkinElmer, Hopkinton, MA, USA). The images were sequentially processed and subjected to 3D reconstruction. CT-AN software was utilized to calculate the parameters.

### Histological analysis

Bones preserved in paraformaldehyde were decalcified with EDTA for approximately four weeks until softened, then embedded in paraffin and sectioned. Following the established methodology, the sections were stained with safranin O-fast green stain kit (Solarbio, China) and immunolabeled with MMP9 polyclonal antibody (1:100, Proteintech, China) and COL2A1 polyclonal antibody (1:100, Proteintech, China). Articular cartilage pathological sections were independently evaluated by two researchers using the Osteoarthritis Research Society International (OARSI) scoring criteria [41, 42]. Images were acquired with a microscope (M8, Precipoint, Germany), and representative images were displayed. The area of positive cells in each immunohistochemical image was quantified using ImageJ.

### Elisa

IL-6, IL-1β, and TNF-α levels in rat joint fluid were detected utilizing commercially available kits (Sinobestbio, China) following the manufacturer's guidelines.

### Quantitative real-time PCR (qRT‒PCR)

RNA extraction was performed utilizing TRIzol reagent (Invitrogen, USA), adhering to the protocol provided by the supplier. After extraction, RNA was converted to complementary DNA (cDNA) employing FastKing gDNA Dispelling RT SuperMix (TIANGEN, Germany). The qRT-PCR experiment was conducted using a real-time PCR apparatus (Bio-Rad, USA). Glyceraldehyde-3-phosphate dehydrogenase (GAPDH) was utilized as the housekeeping gene for normalization. The expression levels of mRNA were quantified using the 2^−ΔΔCT^ technique. Primers were designed following the mRNA sequences acquired from the NCBI database (Table S1).

### Western blot

Proteins were extracted from the samples using RIPA lysis buffer (Beyotime, China) supplemented with PMSF (Beyotime, China). After the protein samples were denatured, they underwent separation through SDS-PAGE and were subsequently transferred to PVDF membranes. These membranes were incubated for 2 h. Subsequently, the membrane underwent incubation with primary antibodies, namely, ETV4 (1:1000, Proteintech, China), SLC7A11 (1:1000, Proteintech, China), GPX4 (1:1000, Selleck, USA), and GAPDH (1:1000, Proteintech, China). The following day, the membranes were washed with TBST (Solarbio, China) and then incubated with Ultra-Polymer Goat Anti-Rabbit IgG (H&L)-HRP (1:5000, Proteintech, China). The immunoreactive bands were visualized using a gel imaging system (Bio-Rad, USA).

### Cell culture

The mouse chondrocyte cell line ATDC5, isolated from mouse teratoma fibroblasts, exhibits rapid growth and is commonly utilized as a model for in vitro chondrocyte studies [43, 44]. ATDC5 cells were procured from the Fu Heng Cell Centre (20210609-01, China) and cultured in a monolayer in DMEM/F12 medium (Gibco, USA) comprising 5% FBS (Thermo, USA) and 1% penicillin/streptomycin (Sigma, USA). The human chondrocyte cell line C28/I2 (BFN60803901, China) was maintained in regular DMEM (Gibco, USA) supplemented with 10% FBS and 1% penicillin–streptomycin [45, 46]. After treating cells with hydrogen peroxide (H_2_O_2_, Sinopharm Chemical Reagent Co., Ltd., China) for 4 h, the concentration of H_2_O_2_ that resulted in cell viability of 60–70% compared to the Control group was designated as the modeling concentration. To further explore the effects of α-KG, various concentrations of α-KG were added to H_2_O_2_-damaged cells. Ferrostatin-1 (Fer-1) is a ferroptosis inhibitor (1 μM, MCE, USA) [47]. Erastin (5 μM, MCE, USA) was used to induce ferroptosis in ATDC5 cells.

### Analysis of cell viability

The cells were cultivated in 96-well plates. Each well received 100 μL of a culture solution with CCK8 reagent (Sigma, USA) (CCK-8: DMEM/F12 = 1:10) and was incubated for 2 h. The absorbance at 450 nm was determined utilizing a microplate reader (Tecan, USA).

### Apoptosis detection through flow cytometry

Apoptosis was detected using an Annexin V-FITC/PI kit (Beyotime, China). Cells were harvested by trypsinization and centrifugation. Following PBS wash and supernatant removal, the cells were resuspended in 195 μL of Annexin V-FITC binding solution supplemented with 5 μL of Annexin V-FITC. After incubation, 10 μL of PI dye was added. Subsequently, the samples were subjected to flow cytometry (Bio-Rad, USA) for apoptosis detection.

### Detection of intracellular ROS accumulation

Alterations in ROS levels were measured utilizing the ROS Assay Kit (Beyotime, China). DCFH-DA was prepared at a final dilution of 1:1000 in serum-free culture medium. The cells were subsequently incubated with this diluted DCFH-DA solution. Following incubation, the cells were washed with serum-free medium to remove excess dye and then subjected to fluorescence microscopy for imaging. The fluorescence intensity of reactive oxygen species was measured using a microplate reader. Following the established protocol, ROS levels in cells were analyzed using a cytometer (Beckman Coulter, China).

### Alcian blue staining

After treatment, the cells were gently rinsed twice with PBS. The cells were fixed, acidified, and stained with Alcian blue staining solution (Solarbio, China). The cells were washed with PBS and subsequently imaged using a microscope (Nikon, Japan). The absorbance of Alcian blue dye at 620 nm was determined by a microplate reader.

### Detection of MDA, SOD, GSH, GSSG, and Fe^2+^

The quantification of malondialdehyde (MDA), superoxide dismutase (SOD), GSH, glutathione disulfide (GSSG), and ferrous ion (Fe^2+^) levels in the examined samples was carried out by employing specific assay kits for each biomarker: an MDA assay kit (Beyotime, China), a SOD assay kit (Beyotime, China), a GSH and GSSG Assay Kit (Beyotime, China), and a kit for determining Fe^2+^ content (Solarbio, China).

### Mitochondrial membrane potential (MMP) measurement

The JC-1 kit (Beyotime, China) was used to assess the MMP [48]. JC-1 staining solution was added to the cells, which were then incubated at 37 °C in the dark for 20 min. After two washes with JC-1 staining buffer, the cells were imaged using a fluorescence microscope (Olympus, Japan). Absorbance at the red (590 nm) and green (530 nm) fluorescence wavelengths in each well was measured with a microplate reader. MMP was determined by computing the ratio between the red and green fluorescence intensities.

### Data analysis

Statistical analyses were performed using GraphPad Prism 9 software (La Jolla, CA, USA). Comparisons between two groups were conducted using the Student's *t*-test. Multiple group comparisons were conducted using one-way ANOVA. Quantitative data were presented as mean ± standard deviation (SD). *P* values < 0.05 indicated statistical significance.

## Results

### Serum metabolomics

**Fig. 1:**
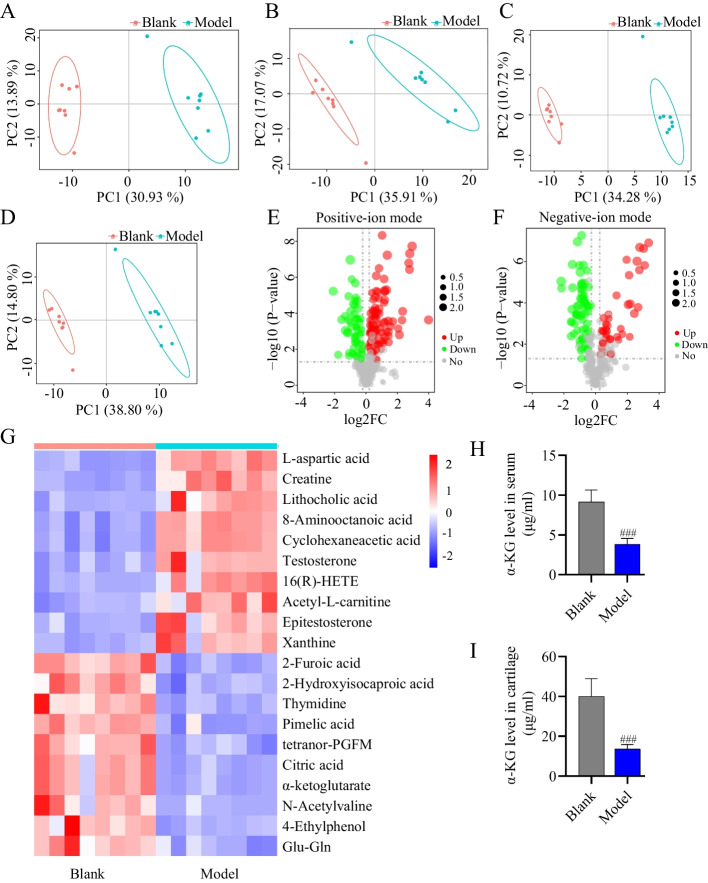
Serum metabolic profiling by LC‒MS in OA rats. **A** PCA score plot of QC analysis in the Blank and Model groups in positive ion mode. **B** PCA score plot of QC analysis in the Blank and Model groups in negative ion mode. **C** Plots of PLS-DA scores in the Blank and Model groups in positive ion mode. **D** Plots of PLS-DA scores in the Blank and Model groups in negative ion mode. **E, F** Volcano plots of metabolites in positive and negative ion modes in the Blank and Model groups. Metabolites significantly expressed are positioned above the dashed line, whereas those not significantly expressed are located below it. **G** Heatmap analysis of differential metabolites: blue bars denote decreased levels, and red bars signify increased levels. **H, I** Levels of α-KG in rat serum and knee cartilage. The statistical significance of the differences between the two groups was assessed using Student's *t*-test. Blank vs Model, ###*p* < 0.001 (*n* = 6)

**Fig. 2:**
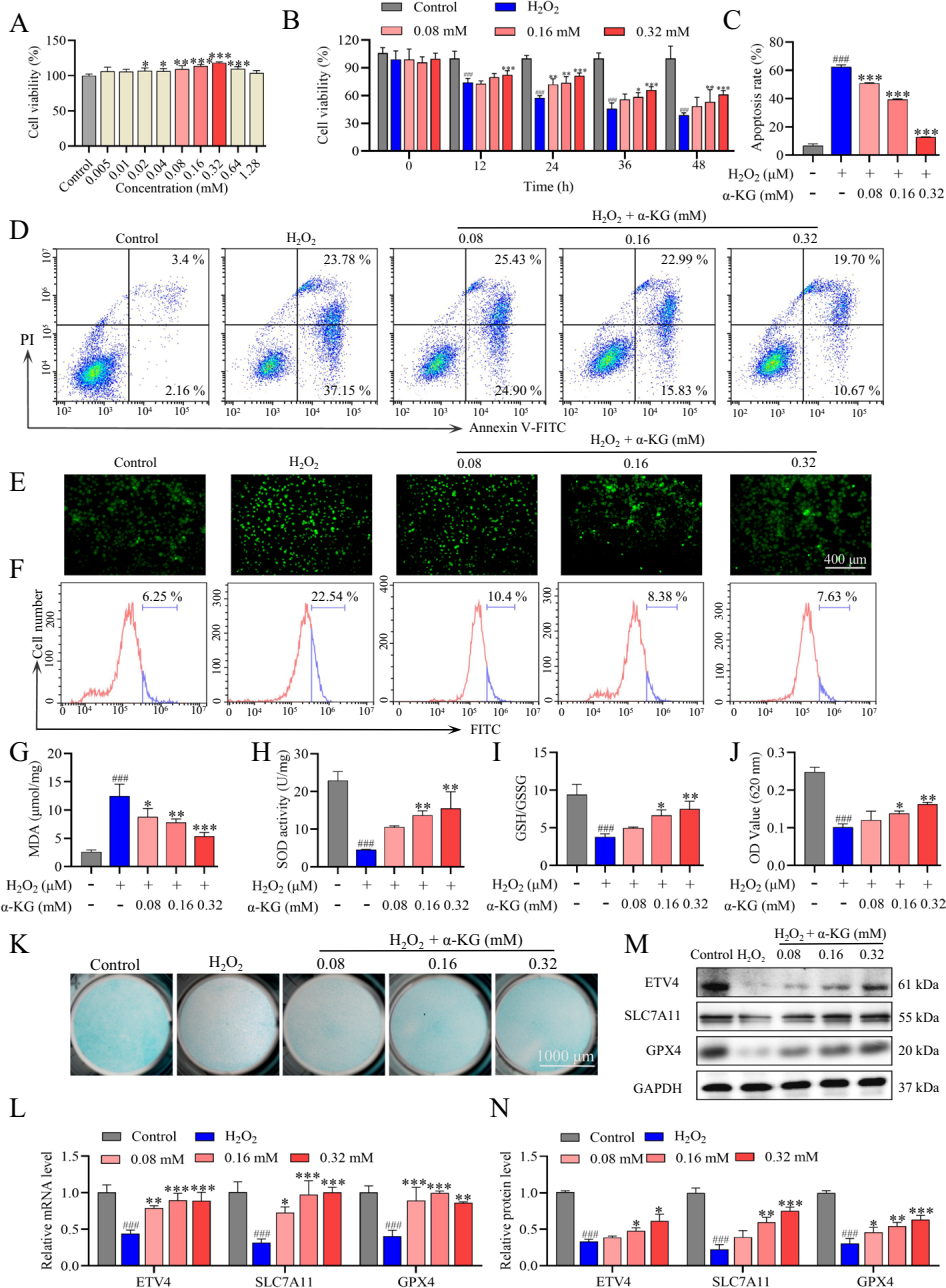
α-KG alleviated oxidative stress and ferroptosis in H_2_O_2_-induced ATDC5 cells.** A** Assessing the changes in cell proliferation 24 h after α-KG treatment. **B** Measuring the effects of different α-KG concentrations on H_2_O_2_-induced ATDC5 cells. **C, D** Flow cytometry was used to assess the apoptosis rate in each group. **E** Fluorescence images of ROS in ATDC5 cells, where the green fluorescence intensity of DCFH-DA represents the ROS level. **F** ROS levels were analyzed using flow cytometry. **G**–**I** Levels of MDA, SOD, and GSH/GSSG in ATDC5 cells. **J, K** Alcian blue quantification and staining. **L** qRT-PCR analysis of ETV4, SLC7A11, and GPX4. **M, N** Western blot analysis and quantification of ETV4, SLC7A11, and GPX4. The statistical significance of the differences among groups was assessed using one-way ANOVA. Control vs H_2_O_2_, ###*p* < 0.001; H_2_O_2_ vs α-KG, *p < 0.05, **p < 0.01, ***p < 0.001 (*n* = 3)

To identify metabolic drivers, LC–MS-based untargeted metabolomics was performed. The PCA results revealed that the quality control (QC) samples of each group gathered well under different ion modes, confirming the repeatability and stability of the method (Fig. 1A, B). PLS-DA revealed significant differences between the Model and Blank groups in both the positive and negative ion modes (Fig. 1C, D), demonstrating that the overall metabolic state of the OA rats was significantly and abnormally altered compared to that of the normal rats. The R2 and Q2 values (R2Y = 1, Q2Y = 0.93 for positive ions; R2Y = 0.99, Q2Y = 0.91 for negative ions) of the PLS-DA score plots demonstrate the model's high fit and reliability, making it suitable for differentially abundant metabolite analysis and screening. The volcano plot further showed a general overview of all the detected serum metabolites (Fig. 1E, F). A total of 264 differential endogenous metabolites were identified in the Model group compared to the Blank group, using the criteria of VIP > 1.0 and *P* < 0.05. Among the 120 metabolites identified in the HMDB database, 56 were upregulated (Table S2) and 64 were downregulated (Table S3). Among these, the top 10 upregulated and downregulated metabolites were selected as potential biomarkers. The upregulated metabolites, potentially serving as diagnostic biomarkers included L-aspartic acid, Creatine, Lithocholic acid, 8-Aminooctanoic acid, Cyclohexaneacetic acid, Testosterone, 16(R)-HETE, Acetyl-l-carnitine, Epitestosterone, and Xanthine. The downregulated metabolites included 2-Furoic acid, 2-Hydroxyisocaproic acid, Thymidine, Pimelic acid, tetranor-PGFM, Citric acid, α-KG, N-Acetylvaline, 4-Ethylphenol, and Glu-Gln, which may serve as disease progression biomarkers and potential therapeutic agents (Fig. 1G). To screen the metabolites with therapeutic potential for OA, 3 hydrophilic metabolites were selected from the downregulated metabolites: Pimelic acid, Citric acid, and α-KG. Through the cell viability test, it was found that α-KG significantly promoted chondrocyte proliferation, while Pimelic acid and Citric acid had little effect on the proliferation of chondrocytes (Figs. 2A, S1A, B). More importantly, the α-KG content in the serum and cartilage was lower in the OA Model group than in the Blank group (Fig. 1H, I). Based on the above analysis, α-KG may be closely related to OA and may have the potential to be an agent for treating OA.

### α-KG alleviated oxidative stress and ferroptosis in H_2_O_2_-induced ATDC5 cells

The CCK-8 assay results revealed that 0.32 mM α-KG was the optimal concentration for ATDC5 (Fig. 2A). Compared with that in the Control group (100%), the activity of ATDC5 cells in the 200 μM H_2_O_2_ group was close to 60–70% (Fig. S2A). Studies have shown that H₂O₂ induces oxidative stress and ferroptosis [49, 50]. Utilizing 200 μM H₂O₂ as the modeling concentration, this study explored the impact of α-KG on H_2_O_2_-induced oxidative stress and ferroptosis in chondrocytes. Compared with those of the H_2_O_2_ group, the cell activities of α-KG groups were significantly higher at 24 h (Fig. 2B). Flow cytometry revealed a significant elevation in the proportion of apoptotic cells in the H_2_O_2_ group, whereas various concentrations of α-KG notably decreased the percentage of apoptotic ATDC5 cells (Fig. 2C, D). The influence of α-KG on cellular antioxidant properties was assessed by measuring ROS levels using the fluorescent probe DCFH-DA. Our findings indicated that stimulating ATDC5 cells with H_2_O_2_ led to the accumulation of intracellular ROS, as evidenced by increased green fluorescence intensity, while α-KG decreased the fluorescence intensity in a concentration-dependent manner (Fig. 2E, S2B). Flow cytometry analysis revealed that the level of ROS in the H_2_O_2_ group increased, but decreased after α-KG treatment (Fig. 2F). H_2_O_2_ treatment alone increased the MDA concentration and reduced both the SOD concentration and the GSH/GSSG ratio in ATDC5 cells. Conversely, α-KG significantly reversed the trends in MDA, SOD, and GSH/GSSG in a dose-dependent manner (Fig. 2G–I). Compared with that in the H_2_O_2_ group, Alcian blue staining revealed an increase in the amount of cartilage matrix in the α-KG-treated ATDC5 cells. Quantitative evaluation further substantiated this result (Fig. 2J, K). Findings suggest that α-KG can enhance ATDC5 cell function by attenuating oxidative damage and ferroptosis.

To investigate whether α-KG has certain clinical application value for treating OA, the effect of α-KG on human chondrocytes (C28/I2) was detected, and the results revealed that 0.16 mM and 0.32 mM α-KG significantly promoted C28/I2 cell proliferation (Fig. S3A). When the H_2_O_2_ concentration was 400 μM, the viability of C28/I2 cells approached 70% of that of the control group. (Fig. S3B). Therefore, 400 μM H_2_O_2_ was chosen as the modeling concentration for C28/I2 cells. In addition, compared with H_2_O_2_ treatment, α-KG treatment at concentrations of 0.16 and 0.32 mM for 24 h significantly increased C28/I2 cell activity (Fig. S3C). The qRT-PCR and Alcian blue staining results revealed that α-KG promoted the expression of genes related to matrix synthesis (COL2A1 and ACAN) and enhanced proteoglycan synthesis in H_2_O_2_-treated C28/I2 cells, suggesting that α-KG could protect human chondrocytes from oxidative damage and has potential for clinical application in the treatment of OA (Fig. S3D‒G).

To further validate the impact of α-KG on H₂O₂-induced ferroptosis in ATDC5 cells, we analyzed the expression of ferroptosis-related genes using qRT-PCR and western blot. H_2_O_2_ inhibited the expression of the ETV4, SLC7A11, and GPX4 mRNAs, and these effects were reversed by α-KG (Fig. 2L). The results of the Western blot analysis were consistent with those of the qRT-PCR (Fig. 2M, N). The data indicate that α-KG activates the ETV4/SLC7A11/GPX4 signaling pathway in ATDC5 cells, which is suppressed by H₂O₂.

### α-KG attenuated Erastin-induced ATDC5 cell apoptosis, oxidative stress, and ECM degradation by inhibiting ferroptosis

**Fig. 3:**
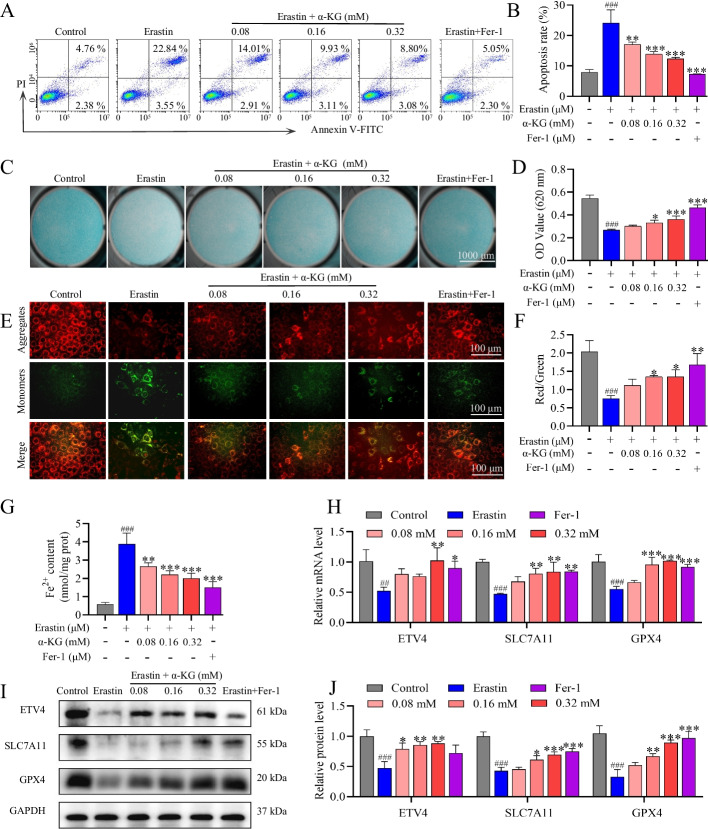
α-KG inhibited ferroptosis in Erastin-induced ATDC5 cells. **A, B** Apoptosis rate of each group. **C, D** Alcian blue staining and quantification. **E** The mitochondrial membrane potential was examined utilizing JC-1. Red, JC-1 aggregates; green, JC-1 monomers. **F** Red fluorescence intensity divided by green fluorescence intensity was used to determine the relative MMP. **G** Fe^2+^ concentration measurement. **H** qRT‒PCR analysis of ETV4, SLC7A11, and GPX4. **I**-**J.** Western blot analysis and quantification of ETV4, SLC7A11, and GPX4. The statistical significance of the differences among groups was assessed using one-way ANOVA. Control vs Erastin, ###*p* < 0.001; Erastin vs α-KG/Fer-1, *p < 0.05, **p < 0.01, ***p < 0.001 (*n* = 3)

Erastin is a prototypical inducer of ferroptosis, exerting its effects by inhibiting cystine uptake mediated by system xc-, which leads to depletion of intracellular GSH and subsequent iron-dependent lipid peroxidation, culminating in cellular ferroptosis [50, 51]. To further investigate the protective role of α-KG against OA through the inhibition of ferroptosis, Erastin was employed to induce cellular ferroptosis. Flow cytometry revealed that the Erastin group exhibited a significant increase in apoptosis, which was markedly reduced by various concentrations of α-KG and Fer-1 (Fig. 3A, B). Alcian blue staining was notably more intense in the α-KG and Fer-1 groups than in the Erastin group, suggesting that α-KG has the potential to inhibit ECM degradation during ferroptosis (Fig. 3C, D). Changes in the MMP were detected via fluorescence microscopy. The ratio of red JC-1 aggregates to green JC-1 monomers decreased with Erastin treatment, and this trend was reversed by α-KG and Fer-1 (Fig. 3E, F). Fe^2+^ levels were significantly greater in the Erastin group than in the α-KG and Fer-1 groups (Fig. 3G). The mRNA expression levels of ETV4, SLC7A11, and GPX4 were significantly lower in the Erastin group than in the Control group. After treatment with different concentrations of α-KG and Fer-1, the mRNA levels were elevated compared with those in the Erastin group (Fig. 3H). The protein levels of ETV4, SLC7A11, and GPX4 were significantly lower in the Erastin group but were increased in the α-KG and Fer-1 groups (Fig. 3I, J). The data indicate that, similar to Fer-1, α-KG treatment alleviates Erastin-induced ferroptosis in ATDC5 cells.

### α-KG relieved pain and alleviated the progression of OA in a rat model

To further explore the role of α-KG-modulated ferroptosis in vivo, we established a rat model of OA.

**Fig. 4:**
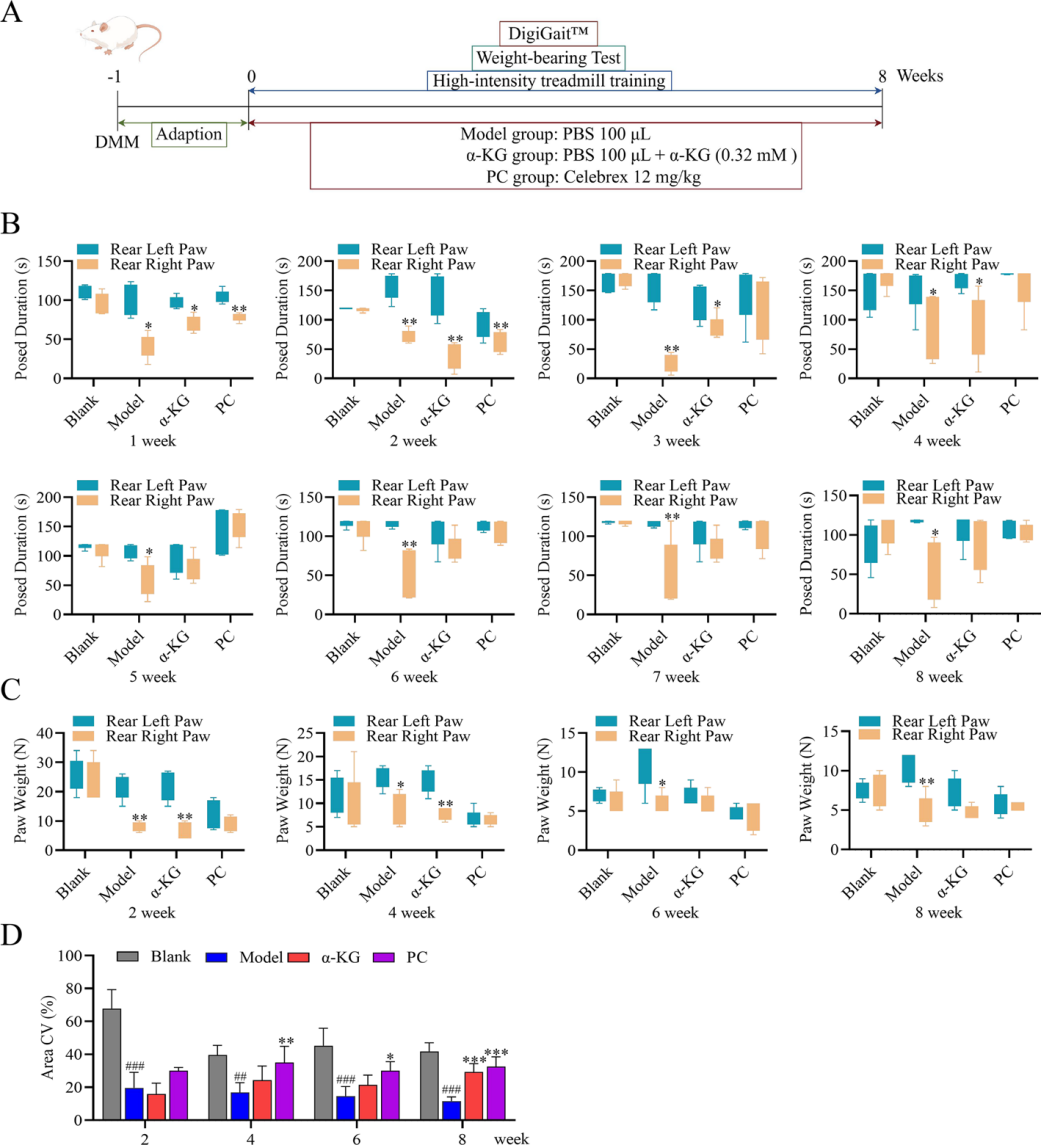
α-KG improved the function of joints in OA.** A** Time plots of the animal experiments (ID: IROIS7887a). **B** Changes in weight-bearing time.** C**, **D** The gait analysis demonstrated the weight-bearing capacity and contact area of the right hind paw. The statistical significance of within-group differences was assessed using Student's *t*-test. Rear Left Paw vs Rear Right Paw, *p < 0.05, **p < 0.01 (*n* = 5). The statistical significance of differences among groups was assessed using one-way ANOVA. Blank vs Model, ##*p* < 0.01, ###*p* < 0.001. Model vs α-KG/PC, *p < 0.05, **p < 0.01, ***p < 0.001 (*n* = 5)

The experimental design is shown in Fig. 4A. One week post-surgery, the DMM rats underwent high-intensity treadmill training five times a week to induce OA development. The right joints of rats in the Model and α-KG groups received weekly injections of PBS or α-KG, respectively. Rats in the PC group were given celebrex daily via gavage. All groups underwent weekly weight-bearing tests and biweekly gait tests. In the first week, significant differences in weight-bearing times between the left and right hind paws were observed in the Model, α-KG, and PC groups; however, these differences were not present in the α-KG group by the fourth week. In contrast, no differences in weight-bearing times were found between the left and right hind paws in the PC group by the third week (Fig. 4B). Gait analysis showed no significant differences in the weight-bearing capabilities of the left and right hind paws between the Blank and PC groups. Two weeks post-modeling, the right hind paw in the model group exhibited a significant reduction in weight-bearing capacity. In the α-KG group, significant differences in hind paw weight-bearing capacity were observed between weeks two and four, with no differences noted between weeks six and eight, indicating that α-KG treatment alleviated pain in the right leg (Fig. 4C). Compared to the Blank group, a significant difference in the contact area of the right hind paw was observed in the model group. In both the α-KG and PC groups, the contact area of the right hind paws decreased, reaching a minimum at week two, and subsequently improved over time (Fig. 4D).

### α-KG improved joint deterioration in OA rats

**Fig. 5:**
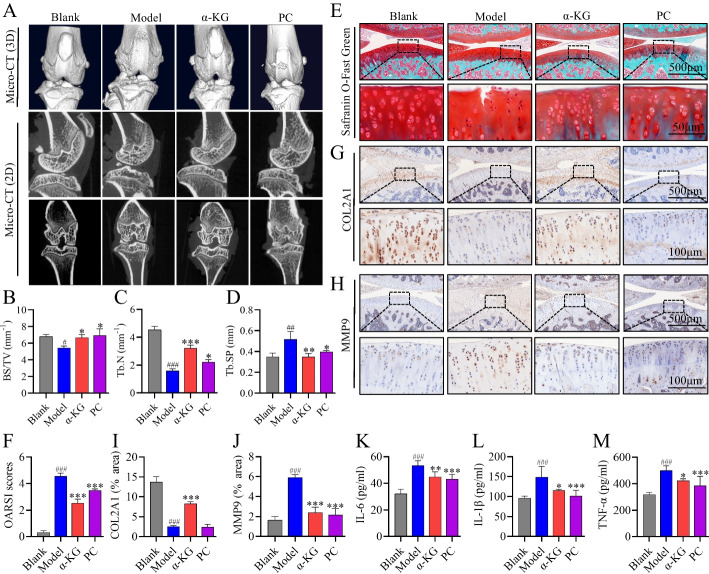
Protection of OA rats by α-KG.** A** Micro-CT three-dimensional reconstructed images and two-dimensional images of knee joints from the different groups. **B**–**D** BS/TV, Tb.N, and Tb.Sp were analyzed by CT-AN software. **E, F** Safranin O-fast green staining and OARSI scores. **G**, **I** IHC staining of COL2A1 and quantitative analysis of the COL2A1-positive area. **H**, **J** IHC staining of MMP9 and quantitative analysis of the MMP9-positive area. **K**–**M** Detection of IL-6, IL-1β, and TNF-α levels in the joint fluid by ELISA. The statistical significance of the differences among groups was assessed using one-way ANOVA. Blank vs Model, #*p* < 0.05, ##*p* < 0.01, ###*p* < 0.001; Model vs α-KG/PC, *p < 0.05, **p < 0.01, ***p < 0.001 (*n* = 5)

The joint structure was first analyzed using Micro-CT. Compared with the Blank group, the Model group had narrower epiphyses, deformed and irregular articular surfaces on sagittal CT images of the knee, significantly lower BS/TV and Tb.n, and greater Tb.Sp. Compared with those in the Model group, the articular surfaces in the α-KG group were more complete. α-KG and celebrex reversed the changes in the BS/TV, Tb.n, and Tb.Sp (Fig. 5A–D). Safranin O-fast green staining revealed disrupted cartilage surface structures, cracks, and burr-like changes in the Model group compared to those in the Blank group. Compared with the Model group, the α-KG and PC groups retained more proteoglycans and exhibited smoother joint surfaces (Fig. 5E). OARSI scores indicated worsened cartilage degradation in the Model group, as evidenced by higher scores than in the Blank group, while the α-KG and PC groups showed reduced scores (Fig. 5F). Immunohistochemistry (IHC) demonstrated decreased COL2A1 levels and increased MMP9 in the model group, whereas α-KG treatment increased COL2A1 levels but showed no significant changes in the PC group. MMP9 expression levels were reduced in both the α-KG and PC groups (Fig. 5G–J). Compared with the Model group, the levels of IL-6, IL-1β, and TNF-α in the joint fluid of the α-KG and PC groups were significantly reduced (Fig. 5K–M).

### α-KG treatment alleviated ferroptosis in the articular cartilage of OA rats

**Fig. 6:**
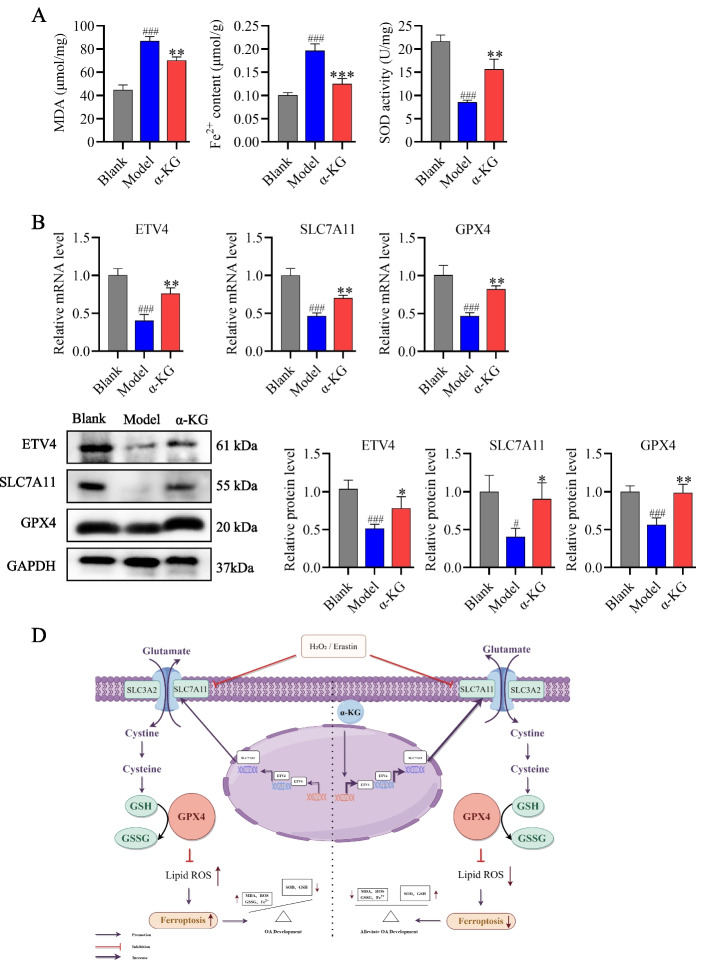
α-KG inhibited ferroptosis by activating the ETV4/SLC7A11/GPX4 pathway.** A** The concentrations of MDA and Fe^2+^, and the activity of SOD in cartilage tissues, were measured. **B** qRT-PCR analysis of ETV4, SLC7A11, and GPX4 in cartilage tissues. **C** Western blot analysis and quantification of ETV4, SLC7A11, and GPX4 in cartilage tissues. **D** A schematic diagram illustrating the mechanism was generated via FigDraw (ID: PISSYcd7bc). The statistical significance of the differences among groups was assessed using one-way ANOVA. Blank vs Model, ###*p* < 0.001; Model vs α-KG, *p < 0.05, **p < 0.01, ***p < 0.001 (*n* = 5)

In the Model group, the levels of MDA and Fe^2+^ increased, whereas the SOD content decreased. α-KG treatment reversed these changes (Fig. 6A). The effect of α-KG on ferroptosis in OA rats was further validated through qRT-PCR and Western blot analyses. The mRNA levels of ETV4, SLC7A11, and GPX4 decreased in the Model group, but this effect was reversed by α-KG (Fig. 6B). Consistent results were observed with Western blot (Fig. 6C). These findings indicate that α-KG attenuates OA by inhibiting ferroptosis in vivo through activation of the ETV4/SLC7A11/GPX4 axis (Fig. 6D).

## Discussion

OA is recognized as the predominant degenerative joint disorder, distinguished by the loss of cartilage, development of osteophytes, and inflammation of the synovial membrane [52]. Although the incidence rate is high, there is currently no medicine available to improve this disease [53]. Serum, serving as the main transporter of small biomolecules within the organism, contains a wide range of biological information. The metabolic signature within the serum is pivotal for delineating both physiological and pathological conditions, facilitating the elucidation of disease mechanisms at the metabolic level. Furthermore, this metabolic profiling enables the detection of early-stage and disease-specific metabolic biomarkers [54–56]. The LC–MS untargeted metabolomics approach was used to screen for significantly different biomarkers in osteoarthritic rats. The top 10 downregulated metabolites could possess therapeutic potential for OA. Among them, 2-Furoic acid, Thymidine, tetranor-PGFM, *N*-Acetylvaline, 4-Ethylphenol, and Glu-Gln exhibit poor water solubility, susceptibility to precipitation, and limited absorption, potentially leading to adverse effects and disruption of biological processes. Despite its water solubility, 2-Hydroxyisocaproic acid is considered hazardous and poses potential risks to the body. To screen the metabolites with therapeutic potential for OA, 3 hydrophilic metabolites were selected from the down-regulated metabolites, including Pimelic acid, Citric acid, and α-KG. Cell viability tests revealed that only α-KG promoted chondrocyte proliferation, while pimelic acid and citric acid had little effect on the proliferation of chondrocytes. Our findings demonstrated a decrease in α-KG levels in both the serum and articular cartilage of OA rats. Therefore, we focused on the mechanism of action of α-KG in OA.

α-KG, a significant intermediate in the tricarboxylic acid (TCA) cycle, has an essential function in facilitating collagen synthesis and expression [57], inhibiting inflammation [58], and preventing oxidative stress [59, 60]. Treatment with H_2_O_2_ can induce ferroptosis and oxidative stress injury in cells [61, 62]. Studies have demonstrated that H_2_O_2_-mediated oxidative stress elevates ROS and MDA levels while reducing GSH/GSSG and SOD in ATDC5 cells, while α-KG can mitigate ATDC5 cell death, reduce ROS and MDA levels, and enhance SOD activity and the GSH/GSSG ratio, thus alleviating oxidative damage in chondrocytes. ROS play a central role in initiating ferroptosis through their involvement in lipid peroxide formation [50]. MDA, a derivative of lipid peroxidation, serves as a marker for oxidative stress and ferroptosis, reflecting the level of damage in lipid membranes [63, 64]. SOD converts superoxide radicals into H_2_O_2_ and oxygen, protecting against oxidative stress [65]. During ferroptosis, saturated antioxidants, including SOD, fail to counteract excessive ROS, resulting in rampant lipid peroxidation [66, 67]. Studies have indicated that ETV4 may function as an inhibitory regulator of various cell death processes, including apoptosis and ferroptosis. ETV4 enhances SLC7A11 transcription by binding to its promoter [68]. SLC7A11, the light chain of system xc-, mediates the exchange of cystine and glutamate across the cell membrane at a 1:1 ratio, promoting GSH synthesis from cysteine [69]. GSH is a key cofactor in peroxide decomposition by GPX4 [70]. GPX4 transforms lipid peroxides into non-toxic alcohols, guarding against lipid peroxidation and playing a crucial role in regulating ferroptosis [71]. ETV4, SLC7A11, and GPX4 constitute important ferroptosis signaling pathways. Our results confirmed that compared to the H_2_O_2_ group, α-KG treatment enhanced the mRNA and protein levels of ETV4, SLC7A11, and GPX4. In summary, our data indicate that α-KG effectively mitigates the impact of ferroptosis on chondrocytes.

Ferroptosis is a novel pattern of cell death characterized by iron dependence and its nonapoptotic nature [50], marked by the accumulation of potentially deadly ROS [72], which has also been found to affect inflammatory diseases [73, 74]. Recent studies have shown that ferroptosis triggers both the onset and progression of OA, including cartilage degradation [75–77]. To elucidate the effects of α-KG on ferroptosis, we induced ferroptosis in ATDC5 cells using Erastin and observed that α-KG and Fer-1 effectively mitigated Erastin-induced apoptosis and ECM degradation. α-KG and Fer-1 upregulated ETV4, SLC7A11, and GPX4 at both the mRNA and protein levels, reducing Fe^2+^ accumulation and preserving the MMP in ATDC5 cells. Collectively, these data suggest that ferroptosis contributes to OA and that α-KG has inhibitory effects on this process.

To further substantiate the protective role of α-KG against OA in vivo, a rat model of OA was established via DMM surgery. α-KG enhanced weight-bearing ability and improved the movement ability of OA rats. Safranin O-fast green staining and IHC staining revealed that α-KG mitigated cartilage degeneration, markedly reducing cartilage erosion and matrix loss in OA. α-KG inhibited the occurrence of ferroptosis in the cartilage of DMM rats by activating the ETV4/SLC7A11/GPX4 pathway. This study also revealed that α-KG can protect human chondrocytes from oxidative damage and has potential for clinical application in treating OA.

## Conclusions

These findings suggest that α-KG can alleviate OA progression by blocking ferroptosis through the ETV4/SLC7A11/GPX4 pathway. Overall, this study provides new insights into the therapeutic potential of α-KG and indicates that it may be a highly promising candidate for OA treatment.

### Supplementary Information

Supplementary Material 1: Fig. S1 Effects of Pimelic acid and Citric acid on ATDC5 cell viability. A. The impact of various concentrations of Pimelic acid on ATDC5 cell viability at 24 h. B. The effects of various concentrations of Citric acid on ATDC5 cell viability at 24 h. The statistical significance of the differences among groups was assessed using one-way ANOVA. Control vs α-KG, ns: not significant (*n* = 5).

Supplementary Material 2: Fig. S2 A. Viability of ATDC5 cells treated with various H_2_O_2_ concentrations. B. ROS fluorescence intensity. The statistical significance of the differences among groups was assessed using one-way ANOVA. Control vs H_2_O_2_, #*p* < 0.05, ##*p* < 0.01, ###*p* < 0.001; H_2_O_2_ vs α-KG, *p < 0.05, **p < 0.01, ***p < 0.001 (*n* = 3).

Supplementary Material 3: Fig. S3 A. The effect of α-KG on the viability of C28/I2 cells at 24 h. B. The viability of C28/I2 cells treated with various H_2_O_2_ concentrations. C. The viability of C28/I2 cells incubated with H_2_O_2_ and α-KG for 24 h. D-E. Alcian blue staining and quantitative analysis. F-G. Gene expression of COL2A1 and ACAN in C28/I2 cells was determined by qRT-PCR analysis. The statistical significance of the differences among groups was assessed using one-way ANOVA. Control vs H_2_O_2_, ##*p* < 0.01, ###*p* < 0.001; H_2_O_2_ vs α-KG, *p < 0.05, **p < 0.01, ***p < 0.001 (*n* = 3)

Supplementary Material 4.

## Data Availability

The data from this study are available from the author for correspondence upon reasonable request.

## Abbreviations

OA: Osteoarthritis
LC‒MS: Liquid chromatography–mass spectrometry
α-KG: α-Ketoglutarate
MDA: Malondialdehyde
SOD: Superoxide dismutase
ECM: Extracellular matrix
ROS: Reactive oxygen species
GPX4: Glutathione peroxidase 4
SLC7A11: Solute carrier family 7 member 11
DMM: Destabilization of the medial meniscus
PBS: Phosphate-buffered saline
H_2_O_2_: Hydrogen peroxide
SD: Standard deviation
PC: Positive Control
PCA: Principal component analysis
PLS-DA: Partial least squares-discriminant analysis
QC: Quality control
ELISA: Enzyme-linked immunosorbent assay
Fer-1: Ferrostatin-1
OARSI: Osteoarthritis Research Society International
cDNA: Complementary DNA
qRT‒PCR: Quantitative real-time PCR
GAPDH: Glyceraldehyde-3-phosphate dehydrogenase
GSH: Glutathione
GSSG: Glutathione disulfide
TCA: Tricarboxylic acid
Fe^2+^: Ferrous ion
IHC: Immunohistochemistry
