# Supplementary figures and images for: α-Ketoglutarate alleviates osteoarthritis by inhibiting ferroptosis via the ETV4/SLC7A11/GPX4 signaling pathway
Source: Cell Mol Biol Lett. 2024 Jun 14;29:88. doi: 10.1186/s11658-024-00605-6 (PMC11177415; doi:10.1186/s11658-024-00605-6)

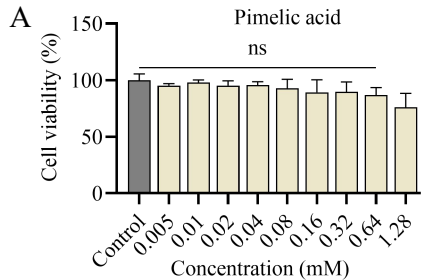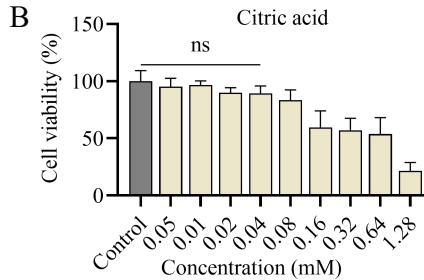

Supplement: Supplementary file 1 — Supplementary Material 1: Fig. S1 Effects of Pimelic acid and Citric acid on ATDC5 cell viability. A. The impact of various concentrations of Pimelic acid on ATDC5 cell viability at 24 h. B. The effects of various concentrations of Citric acid on ATDC5 cell viability at 24 h. The statistical significance of the differences among groups was assessed using one-way ANOVA. Control vs α-KG, ns: not significant (n = 5). [file 11658_2024_605_MOESM1_ESM.pdf]

A

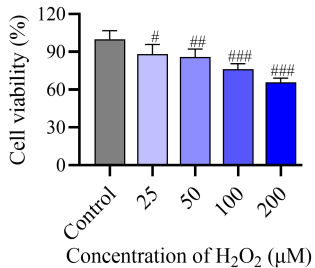

B

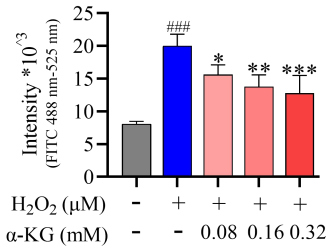

Supplement: Supplementary file 2 — Supplementary Material 2: Fig. S2 A. Viability of ATDC5 cells treated with various H2O2 concentrations. B. ROS fluorescence intensity. The statistical significance of the differences among groups was assessed using one-way ANOVA. Control vs H2O2, #p < 0.05, ##p < 0.01, ###p < 0.001; H2O2 vs α-KG, *p < 0.05, **p < 0.01, ***p < 0.001 (n = 3). [file 11658_2024_605_MOESM2_ESM.pdf]

**A**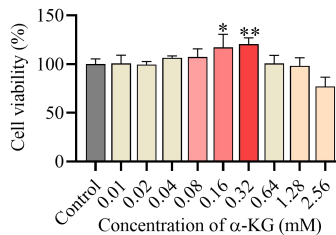**B**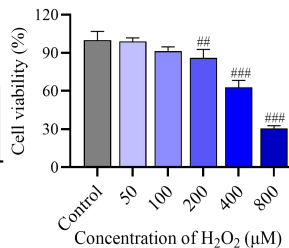**C**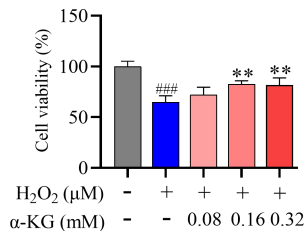**D**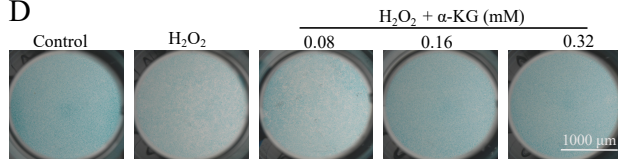**E**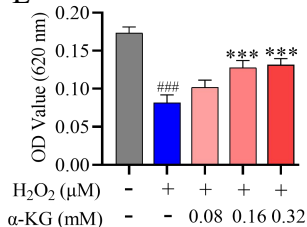**F**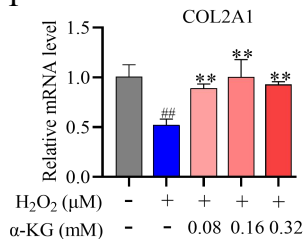**G**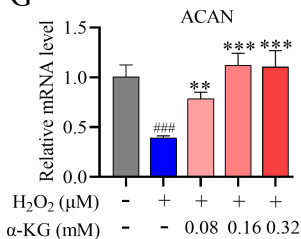

Supplement: Supplementary file 3 — Supplementary Material 3: Fig. S3 A. The effect of α-KG on the viability of C28/I2 cells at 24 h. B. The viability of C28/I2 cells treated with various H2O2 concentrations. C. The viability of C28/I2 cells incubated with H2O2 and α-KG for 24 h. D-E. Alcian blue staining and quantitative analysis. F-G. Gene expression of COL2A1 and ACAN in C28/I2 cells was determined by qRT-PCR analysis. The statistical significance of the differences among groups was assessed using one-way ANOVA. Control vs H2O2, ##p < 0.01, ###p < 0.001; H2O2 vs α-KG, *p < 0.05, **p < 0.01, ***p < 0.001 (n = 3) [file 11658_2024_605_MOESM3_ESM.pdf]
