# Supplementary material for: α-Ketoglutarate alleviates osteoarthritis by inhibiting ferroptosis via the ETV4/SLC7A11/GPX4 signaling pathway
Source: Cell Mol Biol Lett. 2024 Jun 14;29:88. doi: 10.1186/s11658-024-00605-6 (PMC11177415; doi:10.1186/s11658-024-00605-6)
Supplement: Supplementary file 4 — Supplementary Material 4. [file 11658_2024_605_MOESM4_ESM.docx]

**Table.S1** The primer sequences for qRT-PCR

| Primers | Forward (5′ - 3′) | Reverse (5′ - 3′) |
| --- | --- | --- |
| ETV4 (Rat) | GTTTGTGTGTGAGCCAGAGG | CAAGTTCTGGGAGGTAGGCA |
| ETV4 (Mouse) | CAGGTGATGGAGTGATGGGT | TCCAATCCCTTCCTGCTTGA |
| SLC7A11 (Rat) | GGCATGAGAAGGTGGTTCTG | CTGCCCGTGTTCTGGAGTAT |
| SLC7A11 (Mouse) | TGGGCAGGAGAAGGTAGTTC | CTGCCCGTGTTCTGGAGTAT |
| GPX4 (Rat) | GGCAGGAGCCAGGAAGTAAT | TCATCCATTTCCACAGTGGGT |
| GPX4 (Mouse) | CGCCAAAGTCCTAGGAAACG | AACTCGGAGCTGTTGCAGTA |
| COL2A1 (Human) | CATGAGGGCGCGGTAGAG | TCTCCGAAGGGGATCTCAGG |
| ACAN (Human) | AGTCCCTGACCTGGTTTCTG | AGTTCCACAGACCCTAAGCC |
| GAPDH (Rat) | CAAGGCTGAGAATGGGAAGC | GAAGACGCCAGTAGACTCCA |
| GAPDH (Mouse) | AATGTGTCCGTCGTGGATCT | AGACAACCTGGTCCTCAGTG |
| GAPDH (Human) | ACCCAGAAGACTGTGGATGG | TCAGCTCAGGGATGACCTTG |

**Table. S2** List of differentially expressed (upregulated) endogenous metabolites

| Name | HMDB_ID | FC | log2FC | P-value | ROC | VIP |
| --- | --- | --- | --- | --- | --- | --- |
| L-aspartic Acid | HMDB0000191 | 2.444779822 | 1.289704541 | 4.72E-09 | 1 | 1.795007788 |
| Creatine | HMDB0000064 | 2.058767643 | 1.041781013 | 3.20E-07 | 1 | 1.664977145 |
| Lithocholic Acid | HMDB0000761 | 4.305877831 | 2.106307387 | 4.55E-06 | 1 | 1.576946744 |
| 8-Aminooctanoic acid | HMDB0247418 | 3.085362661 | 1.625440077 | 5.12E-06 | 1 | 2.272964636 |
| Cyclohexaneacetic acid | HMDB0031403 | 2.791205357 | 1.480888272 | 5.67E-06 | 1 | 2.316479701 |
| Testosterone | HMDB0000234 | 1.923768726 | 0.94393537 | 6.26E-06 | 1 | 1.265593335 |
| 16(R)-HETE | HMDB0004680 | 2.830028785 | 1.500816727 | 6.32E-06 | 1 | 1.783144469 |
| Acetyl-L-carnitine | HMDB0240773 | 2.046091567 | 1.03287071 | 1.52E-05 | 1 | 1.352534784 |
| Epitestosterone | HMDB0000628 | 2.808430265 | 1.48976398 | 1.84E-05 | 0.984375 | 1.833956812 |
| Xanthine | HMDB0000292 | 2.724328713 | 1.445900787 | 2.59E-05 | 1 | 1.439878017 |
| 8Z,11Z,14Z-Eicosatrienoic acid | HMDB0002925 | 1.65244384 | 0.724601241 | 4.89E-05 | 0.953125 | 1.412295141 |
| Eucalyptol | HMDB0004472 | 1.703285149 | 0.768319979 | 6.84E-05 | 0.96875 | 1.273072192 |
| Hexanoylcarnitine | HMDB0000756 | 1.523562254 | 0.607448451 | 7.85E-05 | 0.984375 | 1.284964448 |
| 5-Phenylvaleric Acid | HMDB0002043 | 1.873678162 | 0.905873165 | 9.22E-05 | 1 | 1.444034045 |
| Choline | HMDB0000097 | 1.291709477 | 0.369281625 | 9.98E-05 | 0.96875 | 1.371613694 |
| Androsterone | HMDB0000031 | 1.660442624 | 0.731567872 | 0.000106254 | 0.984375 | 1.642386808 |
| Uric acid | HMDB0000289 | 1.663883411 | 0.734554347 | 0.000159909 | 1 | 1.561679647 |
| Riboflavin | HMDB0000244 | 1.624229359 | 0.699755372 | 0.000166072 | 0.921875 | 1.264634795 |
| L-Glutamate | HMDB0060475 | 1.451029567 | 0.537076917 | 0.000205423 | 0.984375 | 1.602608505 |
| Pregnanetriol | HMDB0006070 | 7.343806973 | 2.876528139 | 0.000244193 | 0.96875 | 1.57196406 |
| Prostaglandin E1 | HMDB0001442 | 2.82037649 | 1.49588776 | 0.000293981 | 0.953125 | 1.37663732 |
| Chenodeoxycholic Acid | HMDB0000518 | 5.414408745 | 2.436803804 | 0.000328904 | 0.90625 | 1.709554441 |
| 3-Methylcrotonylglycine | HMDB0000459 | 2.72350016 | 1.445461952 | 0.00035805 | 0.984375 | 2.069003636 |
| 13Z,16Z-Docosadienoic Acid | HMDB0061714 | 1.426078904 | 0.512053807 | 0.000532197 | 0.96875 | 1.38600446 |
| Glycolithocholic acid | HMDB0000698 | 1.770299617 | 0.823993552 | 0.000574192 | 0.9375 | 1.025748714 |
| 5-OxoETE | HMDB0010217 | 2.14244547 | 1.099258485 | 0.000656693 | 0.96875 | 1.749683842 |
| Bicyclo prostaglandin E2 | HMDB0249187 | 5.955151985 | 2.574138328 | 0.000706347 | 0.9375 | 1.546768151 |
| Taurine | HMDB0000251 | 1.740796323 | 0.799747415 | 0.000882573 | 0.96875 | 1.753052341 |
| Daidzein | HMDB0003312 | 2.409497058 | 1.268732039 | 0.001176656 | 0.90625 | 1.245517409 |
| Pentadecanoic Acid | HMDB0000826 | 1.64993191 | 0.722406488 | 0.001236017 | 1 | 1.698479853 |
| Kojic acid | HMDB0032923 | 1.351997766 | 0.435092768 | 0.001260586 | 0.953125 | 1.268487707 |
| DL-Norvaline | HMDB0251527 | 1.209645878 | 0.274584763 | 0.001272846 | 0.953125 | 1.040555145 |
| Tetrahydrocortisone | HMDB0000903 | 3.50926286 | 1.811168016 | 0.001771414 | 0.890625 | 1.27409146 |
| D-(+)-Maltose | HMDB0000163 | 2.852560926 | 1.5122577 | 0.002118375 | 1 | 1.513312211 |
| 2,3-Dinor-TXB2 | HMDB0002904 | 1.398057708 | 0.483423913 | 0.002320744 | 0.90625 | 1.125719705 |
| Deoxycholic Acid | HMDB0000626 | 1.639933101 | 0.713636963 | 0.002339622 | 0.9375 | 1.079916543 |
| trans-10-Heptadecenoic Acid | HMDB0244268 | 1.387089558 | 0.472060939 | 0.002458923 | 0.953125 | 1.187121685 |
| Celestolide | HMDB0031867 | 2.684047695 | 1.424410308 | 0.003270188 | 0.875 | 1.120790406 |
| Coenzyme Q2 | HMDB0006709 | 1.368531727 | 0.452628881 | 0.003309095 | 0.921875 | 1.591812166 |
| Sorbic acid | HMDB0256823 | 1.329225986 | 0.410586403 | 0.003879078 | 0.890625 | 1.353146157 |
| Estriol | HMDB0000153 | 1.777734075 | 0.830039533 | 0.004227795 | 0.859375 | 1.025821768 |
| 1-Caffeoylquinic Acid | HMDB0304633 | 2.534054465 | 1.341447533 | 0.004421187 | 0.890625 | 1.238696365 |
| Spermine | HMDB0001256 | 2.621761986 | 1.390536718 | 0.004649163 | 0.875 | 1.293106011 |
| Threonine | HMDB0000167 | 1.255305798 | 0.328038854 | 0.004755653 | 0.875 | 1.336607154 |
| Nonadecanoic acid | HMDB0000772 | 1.557712133 | 0.639428647 | 0.005205605 | 0.921875 | 1.408933317 |
| Sulforidazine | HMDB0042015 | 1.896352326 | 0.92322703 | 0.005330099 | 0.90625 | 1.320174885 |
| Histamine | HMDB0000870 | 1.536481852 | 0.619630727 | 0.005359434 | 0.921875 | 1.152310247 |
| cholesteryl sulfate | HMDB0000653 | 1.88398078 | 0.913784247 | 0.005597867 | 0.890625 | 1.35017405 |
| 7-Ketodeoxycholic acid | HMDB0000391 | 2.708497809 | 1.437492924 | 0.006231687 | 0.875 | 1.134462689 |
| Salicylic acid | HMDB0001895 | 1.290860231 | 0.3683328 | 0.007524498 | 0.84375 | 1.04480434 |
| L-Pyroglutamic acid | HMDB0000267 | 1.388089496 | 0.473100588 | 0.008033779 | 0.921875 | 1.119855261 |
| Troxerutin | HMDB0006083 | 1.335690987 | 0.417586278 | 0.0131689 | 0.828125 | 1.207008556 |
| D-Phenylalanine | HMDB0250791 | 1.538416859 | 0.621446478 | 0.016497139 | 0.890625 | 1.292135318 |
| Asaraldehyde | HMDB0029648 | 1.344462635 | 0.427029661 | 0.016675257 | 0.9375 | 1.18237796 |
| N-Acetylneuraminic acid | HMDB0000230 | 1.412098801 | 0.497841034 | 0.016730248 | 0.828125 | 1.024053166 |
| Azelaic acid | HMDB0000784 | 1.262748043 | 0.336566805 | 0.031421875 | 0.8125 | 1.068058712 |

**Table. S3** List of differentially expressed (down-regulated) endogenous metabolites

| Name | HMDB_ID | FC | log2FC | P-value | ROC | VIP |
| --- | --- | --- | --- | --- | --- | --- |
| 2-Furoic acid | HMDB0000617 | 0.543702679 | -0.879110159 | 5.35E-08 | 1 | 1.784111661 |
| 2-Hydroxyisocaproic Acid | HMDB0000665 | 0.69466499 | -0.525610704 | 2.72E-06 | 1 | 1.351751924 |
| Thymidine | HMDB0000273 | 0.512207306 | -0.965200263 | 3.26E-06 | 1 | 2.185321399 |
| Pimelic acid | HMDB0000857 | 0.571770172 | -0.806492736 | 1.06E-05 | 1 | 1.574601035 |
| tetranor-PGFM | HMDB0258925 | 0.38322623 | -1.383731784 | 1.15E-05 | 1 | 1.567680497 |
| Citric acid | HMDB0000094 | 0.538388235 | -0.893281211 | 1.18E-05 | 0.984375 | 1.774575892 |
| α-ketoglutarate | HMDB0000208 | 0.542434589 | -0.882478919 | 1.39E-05 | 0.984375 | 1.761588871 |
| N-Acetylvaline | HMDB0011757 | 0.225646841 | -2.147861515 | 1.97E-05 | 0.984375 | 1.612372468 |
| 4-Ethylphenol | HMDB0029306 | 0.528833095 | -0.919115629 | 2.14E-05 | 1 | 1.532860891 |
| Glu-Gln | HMDB0028817 | 0.496214499 | -1.010964205 | 3.00E-05 | 1 | 1.81088687 |
| Methyl indole-3-acetate | HMDB0029738 | 0.541021688 | -0.886241667 | 3.64E-05 | 1 | 1.345169136 |
| N6-Succinyl Adenosine | HMDB0255303 | 0.553883651 | -0.85234514 | 3.76E-05 | 0.984375 | 1.899733592 |
| 3-Phenyllactic acid | HMDB0000779 | 0.57184918 | -0.806293395 | 5.29E-05 | 1 | 1.302593392 |
| D-Erythro-sphingosine 1-phosphate | HMDB0000277 | 0.636356288 | -0.652093355 | 5.32E-05 | 1 | 2.015673254 |
| Taurocholic acid | HMDB0000036 | 0.510042702 | -0.971310058 | 6.08E-05 | 0.96875 | 1.467185682 |
| Dihydrocoumarin | HMDB0036626 | 0.534126955 | -0.904745403 | 7.06E-05 | 0.984375 | 1.332218659 |
| Porphobilinogen | HMDB0000245 | 0.62032181 | -0.688911246 | 7.59E-05 | 0.96875 | 1.753733437 |
| Bilirubin | HMDB0000054 | 0.647125844 | -0.627881799 | 0.00010545 | 0.984375 | 1.776033634 |
| Arachidonic acid | HMDB0001043 | 0.829498136 | -0.269689355 | 0.000135663 | 0.984375 | 1.043608658 |
| trans-Cinnamic acid | HMDB0000930 | 0.701022059 | -0.512468253 | 0.00013989 | 1 | 1.227259652 |
| Monoolein | HMDB0254854 | 0.316850293 | -1.658126747 | 0.000145478 | 0.921875 | 1.540760125 |
| Quinoline | HMDB0033731 | 0.732310801 | -0.44947202 | 0.000198669 | 0.953125 | 1.365381562 |
| Stearic Acid | HMDB0000827 | 0.35419813 | -1.497371501 | 0.000204261 | 0.984375 | 1.780119765 |
| 5-Hydroxyindole-3-acetic acid | HMDB0000763 | 0.655769506 | -0.608739279 | 0.000226102 | 1 | 1.068323392 |
| Mesalamine | HMDB0014389 | 0.695817945 | -0.523218208 | 0.00030776 | 1 | 1.364048498 |
| 5'-S-Methyl-5'-thioadenosine | HMDB0001173 | 0.42282676 | -1.241861408 | 0.000315956 | 1 | 1.630099438 |
| Citraconic acid | HMDB0000634 | 0.4349582 | -1.201051332 | 0.000334296 | 0.96875 | 1.514514435 |
| Jasmonic acid | HMDB0032797 | 0.544084691 | -0.87809686 | 0.000351374 | 0.984375 | 1.237079048 |
| 3-Amino-4-methylpentanoic acid | HMDB0245808 | 0.448848889 | -1.155698271 | 0.000361736 | 0.953125 | 1.489661474 |
| Ascorbic acid | HMDB0000044 | 0.424505199 | -1.236145871 | 0.000369235 | 0.96875 | 1.520099772 |
| 5-Methyltetrahydrofolic acid | HMDB0001396 | 0.460080178 | -1.120042795 | 0.000554723 | 0.984375 | 1.914140115 |
| Indole-3-acrylic acid | HMDB0000734 | 0.590440625 | -0.760136107 | 0.00060744 | 0.96875 | 1.107380022 |
| N-Isovalerylglycine | HMDB0245644 | 0.562968455 | -0.828874008 | 0.000655607 | 0.921875 | 1.444377869 |
| Thymine | HMDB0000262 | 0.762916855 | -0.390402258 | 0.00077087 | 0.984375 | 1.423706075 |
| 4-Hydroxycoumarin | HMDB0003654 | 0.34348396 | -1.541685366 | 0.000814025 | 0.953125 | 1.748301841 |
| Biliverdin | HMDB0001008 | 0.320183467 | -1.64302928 | 0.000900862 | 1 | 1.96022153 |
| Deoxycytidine | HMDB0000014 | 0.648952468 | -0.623815282 | 0.000911566 | 0.984375 | 1.492126206 |
| 2'-Deoxycytidine | HMDB0000014 | 0.771566847 | -0.37413694 | 0.001256508 | 0.90625 | 1.012151232 |
| Biotin | HMDB0000030 | 0.71134577 | -0.491377101 | 0.001525694 | 0.9375 | 1.03181295 |
| N-Acetylalanine | HMDB0255053 | 0.571088268 | -0.808214347 | 0.001772495 | 0.953125 | 1.347377909 |
| 3,3',5-Triiodo-L-thyronine | HMDB0000265 | 0.545656684 | -0.873934573 | 0.001821204 | 0.890625 | 1.133125449 |
| 3-Indoxyl sulphate | HMDB0000682 | 0.455075187 | -1.13582317 | 0.002157676 | 0.90625 | 1.376944825 |
| Allantoin | HMDB0000462 | 0.665288941 | -0.587947042 | 0.003176204 | 0.859375 | 1.455456863 |
| 2-(14,15-Epoxyeicosatrienoyl) glycerol | HMDB0013651 | 0.58383897 | -0.776357583 | 0.003604616 | 0.875 | 1.216960645 |
| Glycitin | HMDB0002219 | 0.613825918 | -0.704098531 | 0.003957764 | 0.9375 | 1.40360684 |
| L-Kynurenine | HMDB0000684 | 0.537717093 | -0.895080763 | 0.004019516 | 0.9375 | 1.233823595 |
| N-Tigloylglycine | HMDB0000959 | 0.342392481 | -1.546277075 | 0.004088081 | 0.9375 | 1.57938112 |
| 1-Palmitoyl-Sn-Glycero-3-Phosphocholine | HMDB0010382 | 0.801001757 | -0.320122687 | 0.004358484 | 0.890625 | 1.017969256 |
| Methyl palmitate | HMDB0061859 | 0.728572618 | -0.456855319 | 0.004564119 | 0.90625 | 1.425028446 |
| Kanamycin | HMDB0015303 | 0.601238946 | -0.733989631 | 0.004603372 | 0.90625 | 1.312496214 |
| 3-Methyl-2-oxobutanoic acid | HMDB0030027 | 0.553585071 | -0.853123058 | 0.004857296 | 0.90625 | 1.249010997 |
| Cytosine | HMDB0000630 | 0.827497394 | -0.273173327 | 0.006082679 | 0.921875 | 1.344107044 |
| L-Ascorbic acid 2-sulfate | HMDB0060649 | 0.563457493 | -0.827621315 | 0.006217676 | 0.90625 | 1.299884891 |
| Deoxycorticosterone | HMDB0000016 | 0.502407562 | -0.993069915 | 0.00682792 | 0.875 | 1.540288152 |
| N-Acetyl-L-leucine | HMDB0011756 | 0.658952081 | -0.60175454 | 0.006859631 | 0.859375 | 1.126772696 |
| L-Leucyl-L-Alanine | HMDB0304783 | 0.497615534 | -1.006896572 | 0.008713628 | 0.875 | 1.145597055 |
| 2-Ketohexanoic acid | HMDB0001864 | 0.49872222 | -1.003691612 | 0.009875544 | 0.859375 | 1.17766294 |
| 3-Methylcytidine | HMDB0240577 | 0.785766757 | -0.347826962 | 0.010029393 | 0.859375 | 1.442117532 |
| 2-Hydroxycaproic acid | HMDB0001624 | 0.624132853 | -0.680074941 | 0.013995776 | 0.875 | 1.106090233 |
| Diacetoxyscirpenol | HMDB0035104 | 0.633047102 | -0.659615247 | 0.016027823 | 0.875 | 1.043025703 |
| Adipic acid | HMDB0000448 | 0.71383089 | -0.486345762 | 0.01605064 | 0.859375 | 1.068758399 |
| N-Isobutyrylglycine | HMDB0000730 | 0.702529094 | -0.509370122 | 0.016168616 | 0.875 | 1.100978932 |
| gamma-Glutamylleucine | HMDB0011171 | 0.727058756 | -0.459856138 | 0.041239384 | 0.875 | 1.192139117 |
| Dodecanedioic acid | HMDB0000623 | 0.706324318 | -0.501597327 | 0.046845307 | 0.96875 | 1.146044936 |
| Name | HMDB_ID | FC | log2FC | Pvalue | ROC | VIP |
| 2-Furoic acid | HMDB0000617 | 0.543702679 | -0.879110159 | 5.35E-08 | 1 | 1.784111661 |
